# Systematic evaluation of computational methods for cell segmentation

**DOI:** 10.1093/bib/bbag066

**Published:** 2026-02-24

**Authors:** Rongrong Yang, Guangfu Xue, Zuxiang Wang, Yideng Cai, Wenyi Yang, Jinhao Que, Renjie Tan, Haoxiu Sun, Pingping Wang, Zhaochun Xu, Qinghua Jiang, Wenyang Zhou

**Affiliations:** Center for Bioinformatics, School of Life Science and Technology, Harbin Institute of Technology, 92 Xidazhi Street, Nangang District, Harbin 150001, Heilongjiang Province, China; Center for Bioinformatics, School of Life Science and Technology, Harbin Institute of Technology, 92 Xidazhi Street, Nangang District, Harbin 150001, Heilongjiang Province, China; School of Interdisciplinary Medicine and Engineering, Harbin Medical University, 157 Baojian Road, Nangang District, Harbin 150081, Heilongjiang Province, China; Center for Bioinformatics, School of Life Science and Technology, Harbin Institute of Technology, 92 Xidazhi Street, Nangang District, Harbin 150001, Heilongjiang Province, China; Center for Bioinformatics, School of Life Science and Technology, Harbin Institute of Technology, 92 Xidazhi Street, Nangang District, Harbin 150001, Heilongjiang Province, China; Center for Bioinformatics, School of Life Science and Technology, Harbin Institute of Technology, 92 Xidazhi Street, Nangang District, Harbin 150001, Heilongjiang Province, China; School of Interdisciplinary Medicine and Engineering, Harbin Medical University, 157 Baojian Road, Nangang District, Harbin 150081, Heilongjiang Province, China; School of Interdisciplinary Medicine and Engineering, Harbin Medical University, 157 Baojian Road, Nangang District, Harbin 150081, Heilongjiang Province, China; School of Interdisciplinary Medicine and Engineering, Harbin Medical University, 157 Baojian Road, Nangang District, Harbin 150081, Heilongjiang Province, China; School of Interdisciplinary Medicine and Engineering, Harbin Medical University, 157 Baojian Road, Nangang District, Harbin 150081, Heilongjiang Province, China; Center for Bioinformatics, School of Life Science and Technology, Harbin Institute of Technology, 92 Xidazhi Street, Nangang District, Harbin 150001, Heilongjiang Province, China; School of Interdisciplinary Medicine and Engineering, Harbin Medical University, 157 Baojian Road, Nangang District, Harbin 150081, Heilongjiang Province, China; School of Interdisciplinary Medicine and Engineering, Harbin Medical University, 157 Baojian Road, Nangang District, Harbin 150081, Heilongjiang Province, China

**Keywords:** cell segmentation, nuclei segmentation, image processing, spatial transcriptome, deep learning

## Abstract

Cell segmentation plays a crucial role in elucidating cell structure and function, understanding disease mechanisms, and aiding pathological diagnosis. Current surveys primarily categorize methods by their technical evolution stages, which may not fully capture the paradigm shift brought by deep learning. Moreover, their evaluation scope is largely confined to image-only approaches, overlooking the significant potential of multimodal data in enhancing cell/nucleus segmentation performance. Therefore, we propose a dual-dimensional classification framework for deep learning methods. It categorizes such methods into two types: task-oriented (e.g. semantic or instance segmentation) and data-oriented (e.g. single or multimodal inputs). Based on this, we systematically classify and summarize methods across various segmentation tasks and imaging modalities. We also develop a benchmark test that covers both single-modal and multimodal methods. This test uses five diverse datasets, among which four are from conventional microscopy and one integrates sequencing with image data. Furthermore, it assesses seven algorithms based on three dimensions: effectiveness, robustness, and efficiency. Key findings indicate that deep learning models generally outperform traditional algorithms, with their advantage becoming more pronounced when image data is integrated with sequencing information.

## Introduction

Cell segmentation [1] is crucial to understanding cell growth and differentiation. By analyzing microscopic cell images, it effectively isolates cellular areas from their surrounding environment [2, 3], aiding doctors in observing cellular structures, morphologies, and quantities. With advances in computer vision [4], these techniques have become highly efficient. They now help doctors to quickly and accurately identify abnormal cells during cancer screening, which is important for early detection and intervention [5, 6].

Recent advances in the subcellular imaging transcriptomics [7, 8] have enabled high-resolution spatial mapping of gene expression. While offering new insights into intracellular gene expression, this progress also poses challenges for accurate cell identification and transcript assignment. Spatial omics was named one of the ‘Top 10 Emerging Technologies of 2023’ by the World Economic Forum on June 26, 2023. Spatial transcriptome [9–11] combines imaging, biomarkers, sequencing, and bioinformatics to study biological tissues by revealing cell types, differentiation states, cell–cell interactions, and disease mechanisms. As this technology evolves, more precise cell segmentation techniques are essential to ensure accurate differentiation and identification of cellular components. These improvements are expected to provide reliable and detailed cellular information, advancing medical research and clinical practice.

Given the diversity and complexity of cell segmentation methods, this study provides a comprehensive overview of current key technologies. These methods are systematically divided into three categories: traditional technology-based methods, traditional machine learning-based methods, and deep learning-based methods. Traditional techniques utilize discontinuities and similarities in grayscale values to partition regions and enhance contrasts. Traditional machine learning methods use algorithms such as SVM and K-means for efficient feature extraction. Deep learning-based approaches use neural networks to analyze the real objects corresponding to each pixel in the image. Within the deep learning category, we introduce a classification framework consisting of task-oriented and data-oriented methods. Furthermore, we propose a four-dimensional ‘task-target-modality-method’ taxonomy, addressing the challenge of cross-scenario method comparison.

Owing to the biological significance, high technical feasibility of nuclei, we conducted a systematic evaluation of nuclear segmentation methods. We constructed five independent test datasets, including image-only and image-transcriptomics integrated types, and evaluated seven mainstream tools based on effectiveness, robustness, and efficiency. Our analysis suggests that deep learning will dominate and drive innovation in nuclear segmentation [12–16], while integration with multimodal data such as images and sequencing information will open new possibilities and further advance the field.

##  Overview of cell segmentation methods

Cell segmentation refers to the identification of cellular structures in images, encompassing nuclei, cytoplasm, or whole cells (Table 1). As a broad concept, its precise meaning depends on the context. It underpins key downstream tasks including cell counting, morphological analysis, and dynamic tracking. Nuclei segmentation [17–19] extracts and delineates nuclear contours. Staining often provides high contrast and stable positioning, facilitating accurate localization and serving as a reliable basis for further analysis. Its applications include nuclei counting, nuclei morphological analysis, such as linking size and shape changes to disease. Cytoplasmic segmentation [20, 21] focuses on the region outside the nucleus, which typically exhibits low contrast and varies greatly in shape across cell types (e.g. epithelial sheets or neuronal dendrites). It often builds upon nucleus segmentation results and is used in protein expression analysis, exosome research, and cell migration assessment.

**Table 1:** Differences between nuclear segmentation, cytoplasmic segmentation, organelle segmentation and whole cell segmentation.

Type	Image challenges	Algorithmic difficulties	Marker dependency	Morphological diversity
Nuclear segmentation	Nuclear dense accumulation (e.g. tumor tissue), staining is uneven (e.g. deep and shallow nuclei)	Overlapping/contact nuclei need to be separated (especially nuclear masses in pathological sections)	Depends on staining dyes (e.g. DAPI, Hoechst, hematoxylin)	The nuclear morphology is relatively conservative (but pathological samples may be extremely atypical), mostly oval/spherical
Cytoplasmic segmentation	The signal of the cytoplasm weak (low contrast), which is easily disturbed by adjacent cells	Nuclear and cytoplasmic boundaries of cells	Nuclear and cytoplasmic dual labeling	Irregular, such as dendrites/pseudopodia
Organelles segmentation	Sub-resolution structure is blurred and the signal to noise ratio is low	Subpixel structure detection (e.g. mitochondrial crest)	Specific fluorescent markers are required (e.g. Mito Tracker)	The structure is complex and variable, such as mitochondrial reticulum/lysosomes spots
Whole cell segmentation	Blurred pseudopodia/cell adhesion	The cell membranes in close contact need to be distinguished	Membrane labeling is required (CellMask/WGA)	Cellular morphology varies greatly, such as neurons, epithelial cells

Organelle segmentation [22, 23] targets fine subcellular structures such as mitochondria and the endoplasmic reticulum. Their small size and diverse morphologies require specialized fluorescence labeling and high-resolution imaging (e.g. confocal microscopy). Its applications include organelle quantification, morphology analysis, and dynamic tracking. Whole cell segmentation [24, 25] entails extracting the complete cellular outline, including the nucleus, cytoplasm, and membrane. A core challenge lies in distinguishing closely adhered cells or faint membrane boundaries. It is essential for measuring morphological parameters, population distribution, and drug response evaluation. These tasks vary significantly in resolution requirements, annotation cost, and algorithmic difficulty. Specifically, organelle segmentation requires the highest resolution, cytoplasm segmentation algorithm is the most difficult, and whole cell segmentation has the most expensive annotation cost. These technical challenges have driven the development of diverse computational approaches for cell segmentation.

With ongoing research, numerous cell segmentation methods have been developed. According to their core technical principles and data processing strategies, these methods can be broadly classified into three categories: traditional technology-based, traditional machine learning-based, and deep learning-based approaches (Table 2). Each category has its own developmental history, technical features, and applicable scenarios, making them suitable for different experimental needs and data conditions. The following sections detail each of these three types of methods.

**Table 2:** Overview of cell segmentation methods.

Category	Method	Segmentation object	Imaging modalities	Segmentation paradigm	Strategy	Pipeline/ algorithm/ platform	Programming tool	Reference
Tradition technology	Morphological segmentation	Whole cell	Brightfield microscopy	Instance segmentation	A multi-scale decomposition method based on mathematical morphological operations	algorithm	Python	[26]
		Whole cell	Fluorescence microscopy	Instance segmentation	Iterative masking erosion and dilation	algorithm	Python	[27]
		Nuclear/ Cytoplasmic	Brightfield microscopy	Instance segmentation	Self-dual multi-scale morphology switching operator	algorithm	Python	[28]
	Watershed	Nuclear/ Cytoplasmic	Magnetic resonance/ Brightfield microscopy	Semantic segmentation/ Instance segmentation	Spatial topology based on image gradients	algorithm	Python	[29–36]
	Threshold segmentation	Whole cell / Nuclear/ Cytoplasmic	Brightfield microscopy	Semantic segmentation/ Instance segmentation	Otsu threshold	algorithm	Python	[37–39]
		Whole cell	Brightfield microscopy	Semantic segmentation	Color thresholding	algorithm	Python	[40–42]
	Edge detection segmentation	Unknown	Unknown	Unknown	Canny operator	algorithm	Python	[43]
		Unknown	Unknown	Unknown	Sobel operator	algorithm	Python	[44]
		Unknown	Unknown	Unknown	Laplace operator	algorithm	Python	[45]
		Unknown	Unknown	Unknown	LOG operator	algorithm	Python	[46]
	MINS	Nuclear	Fluorescence microscopy	Instance segmentation	Seeded geodesic	platform	Matlab /C++	[47, 48]
Tradition machine learning	SVM	Organ	Magnetic resonance	Semantic segmentation	The kernel mapping finds the optimal hypersurface	algorithm	Python	[49]
	Random forest	Tissue	Brightfield microscopy	Semantic segmentation	Multi-tree sampling for integrated decision-making	algorithm	Python	[50, 51]
	k-means	Whole cell /Organ	Fluorescence microscopy/ Magnetic resonance	Semantic segmentation	Selective center iterative clustering	algorithm	Python	[38, 52–54]
	ilastik	Whole cell/ Nuclear	Brightfield microscopy/ Fluorescence microscopy	Semantic segmentation	Random forest	platform	Python	[55]
Tradition machine learning	k-NN	Whole cell / Tissue	Brightfield microscopy	Semantic segmentation	Based on proximity tags	algorithm	Python	[56, 57]
	ST-CellSeg	Whole cell	NA	Instance segmentation	Manifold Learning	algorithm	Python	[58]
	ClusterMap	Nuclear	NA	Instance segmentation	Multidimensional scaling	algorithm	Python	[59]
Deep learni ng	U-net	Whole cell	Brightfield microscopy	Semantic segmentation	CNN	algorithm	Python	[60]
	U-net++	Nuclear	Fluorescence microscopy	Semantic segmentation	CNN	algorithm	Python	[61]
	SegNet	Nuclear	Fluorescence microscopy	Semantic segmentation	CNN	algorithm	Python	[62]

(*Continued*)

**Table 2:** Continued

Category	Method	Segmentation object	Imaging modalities	Segmentation paradigm	Strategy	Pipeline/ algorithm/ platform	Programming tool	Reference
	Mask R-CNN	Whole cell	Fluorescence microscopy	Instance segmentation	CNN	algorithm	Python	[63]
	Cell-DETR	Whole cell	Brightfield microscopy	Instance segmentation	Transformer	algorithm	Python	[64]
	CellT-Net	Whole cell	Phase contrast microscope	Instance segmentation	Transformer	algorithm	Python	[65]
	QuPath	Whole cell	Brightfield microscopy	Semantic segmentation and instance segmentation	CNN/ Transformer	Platform	Python	[66]
	CellBin	Whole cell / Nuclear	Brightfield microscopy/ Fluorescence microscopy	Semantic segmentation and instance segmentation	Gaussian Mixture Model	pipeline, platform	Python	[67]
	HoVer-Net	Nuclear	Brightfield microscopy	Instance segmentation	CNN	algorithm	Python	[68]
	CIA-Net	Nuclear	Brightfield microscopy	Instance segmentation	CNN	algorithm	Python	[69]
	Bend-Net	Nuclear	Brightfield microscopy	Instance segmentation	CNN	algorithm	Python	[70]
	nucleAlzer	Nuclear	Brightfield microscopy/ Fluorescence microscopy	Instance segmentation	CNN	algorithm, pipeline	Python	[71]
	MultiStar	Whole cell/ Nuclear	Fluorescence microscopy	Instance segmentation	CNN	algorithm	Python	[72]
	NucleiSegNet	Nuclear	Brightfield microscopy	Instance segmentation	CNN	algorithm	Python	[73]
	Splinedist	Whole cell/ Nuclear	Fluorescence microscopy	Instance Segmentation	CNN	algorithm	Python	[74]
Deep learning	STORM	Nuclear	Fluorescence microscopy	Instance segmentation	CNN	pipeline	Python	[75]
	MSRF-Net	Whole cell/ Nuclear	Brightfield microscopy/ Fluorescence microscopy	Instance segmentation	CNN	algorithm	Python	[76]
	DoNet	Whole cell	Fluorescence microscopy	Instance segmentation	CNN	algorithm	Python	[77]
	XPIWIT	Unknown	Unknown	Neither	Unknown	platform	Python	[78]
	DeepImageJ	Whole cell/ Nuclear	Fluorescence microscopy	Semantic segmentation and instance segmentation	CNN	platform	Python	[79]
	ImJoy	Whole cell/ Nuclear	Brightfield microscopy/ Fluorescence microscopy	Semantic segmentation and instance segmentation	CNN	platform	Python	[80]
	Cellpose	Whole cell/ Nuclear	Brightfield microscopy/ Fluorescence microscopy	Instance segmentation	CNN	algorithm	Python	[81]
	StarDist	Whole cell/ Nuclear	Brightfield microscopy/ Fluorescence microscopy	Instance segmentation	CNN	algorithm	Python	[82]
	nnU-Net	Whole cell / Nuclear/ Cytoplasmic/ Organelles	Brightfield microscopy/ Fluorescence microscopy	Semantic segmentation	CNN	algorithm	Python	[83]
	DeepMIB	Whole cell / Nuclear/ Cytoplasmic/ Organelles	Brightfield microscopy/ Fluorescence microscopy	Instance segmentation	CNN	platform	Matlab	[84]
	InstantDL	Nuclear	Brightfield microscopy/ Fluorescence microscopy	Semantic segmentation and instance segmentation	CNN	pipeline, platform	Python	[85]

(*Continued*)

**Table 2:** Continued

Category	Method	Segmentation object	Imaging modalities	Segmentation paradigm	Strategy	Pipeline/algorithm/platform	Programming tool	Reference
CellSAM	Whole cell	Brightfield microscopy	Instance segmentation	Transformer and CNN	algorithm	Python		[86]
DTA-Net	Nuclear	Magnetic resonance	Instance segmentation	Transformer and CNN	algorithm	Python		[87]
Holy-Net	Nuclear	Brightfield microscopy	Instance segmentation	CNN	algorithm	Python		[88]
Mesmer	Whole cell / Nuclear	Brightfield microscopy	Instance segmentation	CNN	algorithm	Python		[89]
Deep learning	DeepCell	Whole cell / Nuclear	Brightfield microscopy/ Fluorescence microscopy	Semantic segmentation and instance segmentation	CNN	pipeline, platform	Python	[90]
	CP-Net	Whole cell / Subcellular	Brightfield microscopy	Instance segmentation	CNN	algorithm	Python	[91]
	MA-Net	Nuclear	Computed Tomography	Semantic segmentation	CNN	algorithm	Python	[92]
	CellSeg	Nuclear	Fluorescence microscopy	Instance segmentation	CNN	algorithm	Python	[93]
	CellProfiler	Whole cell / Nuclear	Brightfield microscopy/ Fluorescence microscopy	Neither	Hybrid integration	platform	Python	[94]
	REU-Net	Nuclear	Brightfield microscopy	Semantic segmentation	CNN	algorithm	Python	[95]
	Mobile-CellNet	Whole cell	Brightfield microscopy	Semantic segmentation	CNN	algorithm	Python	[96]
	Bering	Whole cell	Fluorescence microscopy	Instance segmentation	GNN	algorithm	Python	[97]
	NuSeT	Nuclear	Fluorescence microscopy	Semantic segmentation and instance segmentation	CNN	platform	Python	[98]
	Baysor	Whole cell / Nuclear	Fluorescence microscopy	Instance segmentation	Probabilistic graphical model	algorithm	Python	[99]
	JSTA	Whole cell / Nuclear	Fluorescence microscopy	Semantic segmentation and instance segmentation	DNN	algorithm	Python	[100]
	SCS	Whole cell / Nuclear	Fluorescence microscopy	Instance segmentation	Transformer	algorithm	Python	[101]
	StereoCell	Nuclear	Fluorescence microscopy	Instance segmentation	CNN	platform	Python	[102]
	STCellbin	Whole cell	Fluorescence microscopy	Instance segmentation	CNN	algorithm	Python	[103]
	CelloType	Whole cell	Fluorescence microscopy	Instance segmentation	Transformer	algorithm	Python	[104]
	BIDCell	Nuclear	Fluorescence microscopy	Instance segmentation	CNN	algorithm	Python	[100]
	GeneSegNet	Whole cell / Nuclear	Fluorescence microscopy	Instance segmentation	CNN	algorithm	Python	[105]
	CellSighter	Whole cell	Fluorescence microscopy	Semantic segmentation	CNN	algorithm	Python	[106]
Deep learning	Cellvit	Nuclear	Brightfield microscopy	Instance segmentation	Transformer	algorithm	Python	[107]
	SpiDe-Sr	Nuclear	Fluorescence microscopy	Instance segmentation	CNN	algorithm	Python	[108]

###  Traditional technology-based cell segmentation methods

Traditional technology-based cell segmentation methods are mainly based on threshold, region, edge, and specific theory segmentation methods. These methods are based on the discontinuity and similarity of the gray value of the image, and realize image segmentation by dividing the image into regions with consistent features and the differences between regions.

#### Morphological segmentation

Morphological segmentation [109, 110] is a traditional cell segmentation method that focuses on processing shape features in images and is particularly important for segmenting adherent cells. It employs operations such as dilation, erosion, opening, and closing. These operations remove noise, fill holes, and separate clustered cells to improve image quality, which facilitates accurate boundary delineation. Oliver Schmitt [26] proposed a multiscale morphological decomposition method for cell segmentation in bright-field microscopy images. Wang et al. [27] developed an iterative masking approach using erosion and dilation to segment bacterial, yeast, and mammalian cells, which refines uncertain regions through successive iterations. Dorini LB [28] introduced a self-dual multiscale morphology operator to enhance gradients and edges, improving segmentation in leukocyte images.

Watershed segmentation [29], which interprets a grayscale image as a topographic surface using local minima as basins and their edges as watershed lines for effective partitioning. This method was initially proposed by Digabel and Lantuejoul and later extended by Lantuejoul and Beucher [111]. It is widely applied in cell image segmentation and analysis [112, 113]. When processing cell images with complex backgrounds, watershed segmentation can suffer from reduced accuracy due to noise and difficulty distinguishing cells with subtle morphological differences. The method is also sensitive to parameter choices, which can significantly compromise results. Future efforts could focus on developing more adaptive algorithms with intelligent parameter tuning to overcome these limitations and enable deeper study of cellular structures and functions.

####  Threshold segmentation

Threshold segmentation [114] is a region-based method that plays an important role in nuclei segmentation and recognition by grouping pixels into categories according to feature thresholds. It is particularly suitable for distinguishing objects from background based on gray-level differences, serving as a fundamental preprocessing step in image analysis. Otsu [115], proposed by Nobuyuki, is widely used in image threshold segmentation. It separates an image into background and target classes by maximizing inter-class variance based on gray values. Extensions of this approach include multi-level thresholding by Omer and Elfadi [116], which achieved 96% accuracy in breast segmentation. An adaptive method by Selvamurugan and Sundararajan [117] that combines coarse fuzzy C-means segmentation with fine window-adaptive thresholding for breast cancer detection.

Threshold segmentation is valued for its simplicity and efficiency, yet it has limitations. It often leads to over-segmentation or under-segmentation in challenging conditions such as non-uniform grayscale, uneven lighting, or low target-background contrast. This is particularly problematic in medical imaging, where complex tissue structures challenge accurate organ extraction. Furthermore, by relying solely on grayscale values and ignoring spatial context, the method often fails to capture precise contours and details in complex scenes. Integration with advanced technologies may enable threshold segmentation to overcome these constraints and offer more powerful solutions in complex image analysis.

###  Edge detection segmentation

Edge detection segmentation [118] relies on identifying step or roof-shaped changes in gray scale values at object boundaries, typically by computing the image gradient through convolution with various operators. Common edge detection operators include Canny operator [43], Sobel operator [44], Laplace operator [45] and LOG operator [46]. To enhance edge localization, researchers have developed improved variants. Chia-Jui Hsieh et al. [119] employed an enhanced Canny operator to improve edge contrast and reconstruction quality. KONG W et al. [120] developed a genetic algorithm-based adaptive threshold Sobel method that outperformed conventional edge detection. However, edge detection is highly sensitive to noise, which can introduce false edges, while denoising filters may blur genuine boundaries. Additionally, most methods focus solely on object contours and overlook internal structure details. Algorithms performance also varies across image types, with few single algorithms performing optimally across all scenarios. Combining multiple edge detection algorithms can improve accuracy and robustness, and integrating techniques like region growing or morphological operations may yield more complete segmentation results.

In conclusion, traditional technology-based methods rely on handcrafted features, enabling efficient processing and rapid feature extraction in simple images. However, they struggle with complex cell morphologies, varied backgrounds, and cell adhesion, often failing to capture subtle distinctions and leading to reduced accuracy and robustness. Moreover, their susceptibility to image noise further compromises segmentation quality, limiting their suitability for high-precision applications.

###  Traditional machine learning-based cell segmentation methods

With the rapid technological advancement, machine learning has profoundly influenced cell segmentation [121], introducing innovative approaches and expanding research directions. For instance, Akselrod-Ballin et al. [49] developed a multi-scale, multi-channel method combined with Atlas-based SVM for 3D MR brain images segmentation. Janardhanaprabhu and Malathi [122] proposed a graph-based depth-first search method that organizes pixels into a tree structure, reducing computational complexity and improving accuracy in brain tumor detection compared with ANFIS and SVM. Clustering techniques such as K-means, fuzzy C-means, and SOM group pixels based on features like density but are prone to local optima and limited convergence. Although widely used in medical imaging [123–126], fuzzy C-means often shows constrained adaptability. Rajan and Sundar [52] integrated K-means with FCM and active contours for brain tumor segmentation, demonstrating superior performance in evaluating tumor regions.

Overall, the effectiveness of traditional machine learning-based segmentation hinges on the extraction and design of image features. However, selecting task-specific features or combinations increases training complexity, often resulting in limited performance in challenging scenarios.

###  Deep learning-based cell segmentation methods

####  Task-oriented cell segmentation methods

Semantic segmentation and instance segmentation are two distinct but related computer vision tasks, differentiated primarily by their annotation granularity. Semantic segmentation classifies pixels by category, whereas instance segmentation also differentiates between individual objects within the same category. In biomedical image analysis, particularly in cell segmentation, deep learning methods have demonstrated superior performance over traditional and classical machine learning approaches. Through end-to-end feature learning, deep models adapt better to morphological diversity and complex conditions. This section will focus on deep learning-based cell segmentation methods and discuss the specific applications and strengths of both semantic and instance segmentation in the field.

#####  Semantic segmentation

Semantic segmentation classifies each pixel in an image into a category. A major breakthrough was achieved by Jonathan Long et al. [127] with the Fully Convolutional Networks (FCNs), which replaces fully connected layer with a convolutional layers to enable end-to-end pixel-wise prediction. By incorporating skip connections between deep and shallow layers, FCN combines semantic and fine-grained appearance information to generate precise segmentation outputs.

In medical imaging, Hatipoglu et al. [128] introduced a multi-scale sliding window CNN that outperformed traditional KNN and SVM methods. Similarly, Song et al. [30] proposed a multi-scale CNN (MSCN) for segmenting nuclei and cytoplasm with high accuracy. Ronneberger et al. [60] developed U-Net, which combines upsampling and downsampling paths with skip connections to enable full-image segmentation in a single forward pass. It has become a foundational model in biomedical image segmentation and inspired extensions such as V-Net by Milletari et al. [129], which uses 3D convolutions and a dice-based objective function for volumetric data.

Despite these advances, semantic segmentation still struggles with generalizing across highly variable and complex medical images, particularly under noise and irregular morphology. Future efforts may focus on improving robustness, reducing computational cost, and incorporating transfer learning or few-shot learning to handle diverse and complex scenarios.

#####  Instance segmentation

Instance segmentation detects and distinguishes individual objects within an image. A foundational deep learning-based method is Mask R-CNN [63], which extends Faster R-CNN by adding a segmentation branch to classification and regression. Its first stage uses a Region Proposal Network (RPN) to generate object proposals, while the second stage predicts categories, refines bounding boxes, and employs FCN to segment each instance. Although developed for natural images, Mask R-CNN has been adapted to biomedical tasks. Hollandi et al. [71] introduced nucleAlzer for nuclear instance segmentation, and Eiji Mitate [130] applied it to segment nuclei in oral cell preparations.

Recently, Transformer-based models have gained traction. Tim Prangemeier [64] proposed Cell-DETR, an end-to-end instance segmentation method based on DETR that outperformed Mask R-CNN and U-Net on biomedical data. Wan Z et al. [65] developed CellT-Net using Swin Transformer backbone for multi-scale feature learning, achieving state-of-the-art results. Other innovations include a multi-branch U-shaped transformer by Qiao J et al [131], a parasitic GAN by Sun et al. [132], and semi-supervised regularization techniques by Li et al. [133], collectively enriching the toolbox for nuclei segmentation.

Biomedical images pose distinct challenges due to complex morphology, tissue variability, and noise. Many models require high computational resources and struggle with large, high-resolution images. Future work should focus on integrating multimodal data, improving robustness and efficiency, and enhancing model interpretability for clinical trust.

####  Data-oriented cell segmentation methods

Under the deep learning framework, cell segmentation methods are data-driven, and their performance is ultimately limited by the quality and diversity of the input data. The informational dimension of the data not only shapes the model’s ability to capture morphological features but also its generalization across heterogeneous biological samples. Therefore, it is valuable to classify these methods by data type in addition to their task objectives. Distinctions such as image-based versus sequencing-integrated approaches are key to evaluating their characteristics and applicability.

#####  Image-based cell segmentation me**t**hods

This method focuses on 2D visual information for segmentation. It leverages CNN’s powerful feature-learning capability to extract and analyze texture, shape, color, and other cellular features, enabling accurate end-to-end segmentation. DeepCell [90] integrate tasks like cell and nuclear segmentation along with tracking, employing deep watershed and panoptic models for semantic and instance segmentation. StarDist [82] models nuclear morphology using star-convex polygons based on radial distance probabilities, which is effective for nuclei identification. To address its limitations with non-star-convex structures, Splinedist [74] introduces parametric splines for improved shape modeling. Cellpose [81], a generalist model based on U-Net, produces masks and flow fields without manual parameter tuning, enabling robust whole-cell and nuclear segmentation.

While these algorithms significantly advance biomedical image analysis, several limitations remain. Performance may degrade in complex environments such as diseased cells with abnormal morphology or dense cell interactions. High computational costs also limit deployment in resource-constrained settings. Moreover, most methods focus solely on image features without incorporating biological context such as physiological states or molecular signals, restricting molecular-level analysis. Future developments should emphasize multimodal data integration, lightweight models, and improved interpretability to better support biomedical and clinical applications.

#####  Image- and sequencing-based cell segmentation methods

This method integrates 2D images with sequencing data to emphasize molecular characteristics for accurate cell segmentation. By combining gene expression profiles with image features through a multi-head attention mechanism, it achieves effective multi-source data fusion. This approach demonstrates strong performance in tumor heterogeneity studies, enabling precise cell boundary delineation and differentiation of cancer cell subtypes based on molecular profiles, thereby supporting precision treatment strategies.

SCS [99] tackles cell segmentation in high-resolution spatial transcriptomics by integrating transcriptomic and imaging data with Transformer models to learn complex spatial relationships. In a self-supervised manner, BIDCell [100] combines bioinformatics-aware loss functions to directly learn expression-morphology associations from images without manual annotation. The method improves segmentation accuracy, increases expression purity, and reduces neighbor contamination across diverse platforms and tissue types. CellBin [67] provides an integrated workflow for high-resolution spatial transcriptomic data, excelling in user-friendly image stitching and molecular labeling by leveraging both image and spatial information. GeneSegNet [105] extends the U-Net architecture by incorporating RNA spatial locations and employs recursive training to enhance robustness, enabling effective handling of large images and facilitating segmentation in RNA *in situ* hybridization data.

Despite their strengths, these methods face challenges such as high-dimensional data sparsity, difficulties in feature alignment during fusion, and high computational resource requirements. Performance is often constrained by input data quality and complex parameter tuning, which demands expert knowledge. As spatial omics technologies evolve, further validation of the adaptability and generalization of these tools remains essential. In summary, while existing tools show unique advantages, significant improvements are still needed. Future progress will rely on interdisciplinary collaboration integrating biology, computer science, and mathematics to develop more accurate, efficient, and universally applicable cell segmentation methods, ultimately advancing biomedical research.

##  Benchmarking the performance of nuclei segmentation methods

As hubs of genetic regulation, nuclear morphology and distribution reflect cellular functional states. Therefore, segmenting nuclei is essential for single-nucleus sequencing and spatial transcriptomics, and has become a central focus in the field [134–137]. Based on this importance, we benchmarked nuclei segmentation methods.

To establish a rigorous and practical framework, algorithms were selected against four criteria. These included extensive citation and recognition, multi-modality compatibility, rigorous validation, and unique advantages. Guided by these criteria, we evaluated seven representative algorithms: Watershed [138](classical standard), Cellpose [81](versatile and interactive), StarDist [82](oriented nucleus segmentation), DeepCell [90](high-throughput analysis), Splinedist [74](shape-constrained), Mesmer [89] (multi-channel fusion), and GeneSegNet [105](multi-omics integration). These methods extensively validated [139–146], open-source methods with available pre-trained models span the evolution from classical to deep learning approaches, ensuring comparability and supporting a reproducible evaluation system. The selected methods were divided into two categories by input data type: image-only and image-sequencing integrated. Independent and comparative analyses were then conducted to assess their adaptability to these distinct data characteristics.

###  Performance comparison of different methods on image-only datasets

In this study, we systematically compared six nuclei segmentation methods: Cellpose [81], StarDist [82], DeepCell [90], Splinedist [74], Mesmer [89] and Watershed [138]—across four microscopic image datasets (detailed in Section 5.1). These include: PanNuKe [147], comprising whole-slide images with 189 744 annotated nuclei across 19 tissue types. MoNuSeg [148], containing expert-annotated nuclei from seven organs totaling over 21 000 instances. BBBC041Seg [149], which includes 1328 Giemsa-stained blood smear images with 93 539 leukocyte nuclei, and excludes images containing non-nucleated cells (retaining only samples with leukocyte nuclei). BSST265 [150], featuring 79 fluorescence-DAPI images with 7813 nuclei. All methods performed instance segmentation, and were evaluated directly based on predicted object masks. We adopted several widely used evaluation metrics [151–155], including Intersection–Union Ratio (IoU) for region overlap, Precision for prediction reliability, Recall for detection sensitivity, F1-score for the balance between Precision and Recall, and Dice Similarity Coefficient (Dice) for pixel-level mask consistency. Further details on calculations are provided in Section 5.3.

**Figure 1:**
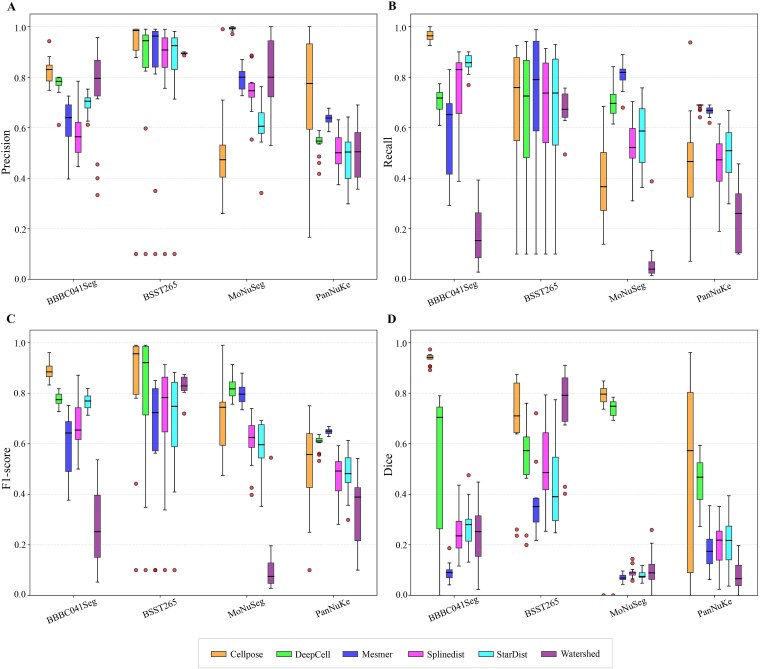
Quantitative comparison of six nuclei segmentation algorithms Cellpose, DeepCell, Mesmer, Splinedist, StarDist and watershed evaluated on four image-only datasets BBBC041Seg, BSST265, MoNuSeg and PanNuKe. (A–D) present the results for precision, recall, F1-score, and dice, respectively. Each boxplot illustrates the distribution of metric values for a given algorithm and dataset, while each point represents the metric value for one image of that dataset.

Effectiveness evaluation across datasets revealed variations in performance (Fig. 1). Cellpose demonstrated strong overall performance, achieving top scores on BBBC041Seg in all key metrics, and excelling in Dice on both BSST265 and MoNuSeg. However, it showed moderate Precision and Recall on MoNuSeg, indicating a trade-off between pixel-level accuracy and instance-level detection. DeepCell and Mesmer relatively excelled in instance-level metrics (Precision, Recall, F1-score) on MoNuSeg and PanNuKe, reflecting robust recognition and balanced error control. DeepCell also performed well in pixel accuracy on MoNuSeg, but suffered from low Recall on BBBC041Seg, leading to suboptimal balance. StarDist and Splinedist comprised a middle tier. StarDist achieved high Recall on BBBC041Seg but low Dice, suggesting boundary errors. Both delivered moderate instance-level performance on MoNuSeg. Splinedist consistently ranked mid-to-low across most datasets. The conventional Watershed method underperformed broadly. While moderately useful for pixel accuracy on BSST265, it exhibited low Recall on BBBC041Seg and generally lagged behind deep learning-based approaches in both instance-level balance and pixel accuracy.

**Figure 2:**
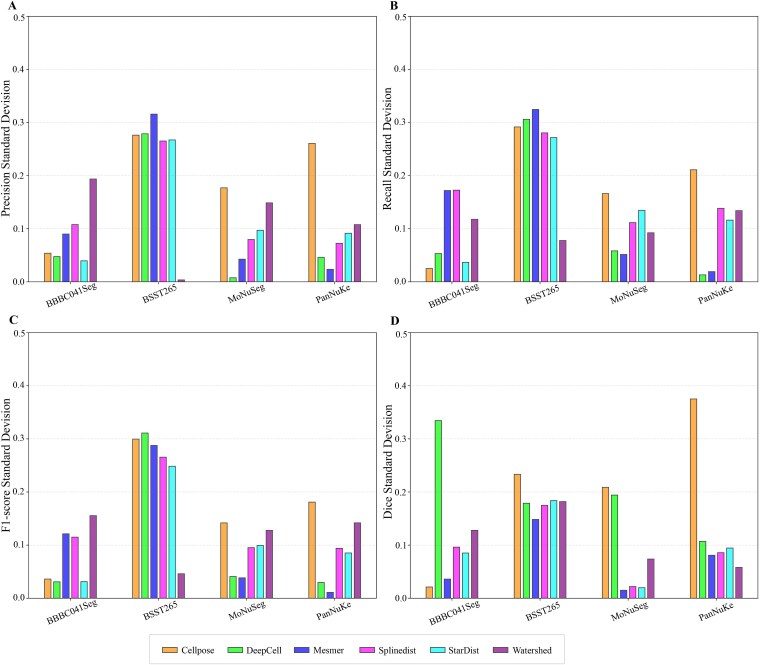
Evaluates the stability of six nuclei segmentation algorithms across four image-only datasets, which include BBBC041Seg, BSST265, MoNuSeg, and PanNuKe, by measuring the standard deviation of their performance metrics. (A–D) present the standard deviations for precision, recall, F1-score, and dice, respectively. Lower values indicate greater algorithmic stability on the corresponding dataset and metric.

**Figure 3:**
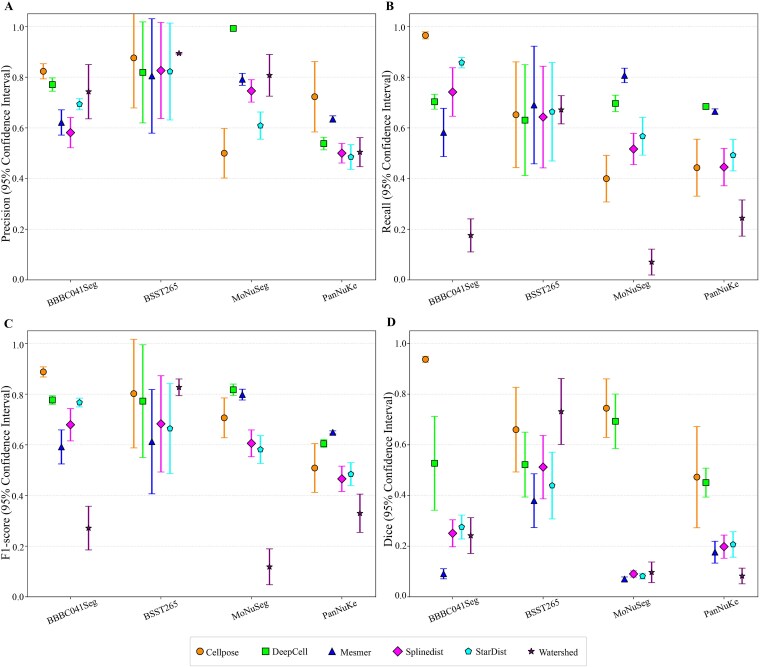
Evaluates the stability of six nuclei segmentation algorithms across four image-only datasets, which include BBBC041Seg, BSST265, MoNuSeg, and PanNuKe, using confidence intervals of performance metrics. (A–D) present the confidence interval for precision, recall, F1-score, and dice, respectively. Narrower confidence intervals indicate better algorithmic performance for the corresponding dataset and metric.

Robustness analysis via standard deviation and confidence intervals highlighted further differences (Fig. 2 and 3). Cellpose and StarDist showed stable performance with low standard deviations and narrow confidence intervals on uniform datasets, owing to morphological priors and high compatibility with regular data. DeepCell and Mesmer displayed consistent results on tissue images, benefiting from multi-scale feature extraction and tissue-specific optimizations. All methods, however, showed variability on the heterogeneous BSST265 dataset, indicating a shared challenge in generalizing to highly diverse data. To investigate the underlying reasons for this phenomenon, we analyzed the original construction and characteristics of the dataset [101]. The results suggest the challenges are likely attributable to inherent, multi-dimensional heterogeneity within the dataset. Specifically, it aggregates samples from ganglioneuroblastoma, Wilms’ tumor, and various cell lines, leading to significant variations in nucleus size, morphology, and texture. Concurrently, fluctuations in sample preparation, imaging modalities, and magnification exacerbate issues such as staining variability, resolution differences, and inconsistent signal-to-noise ratios.

**Figure 4:**
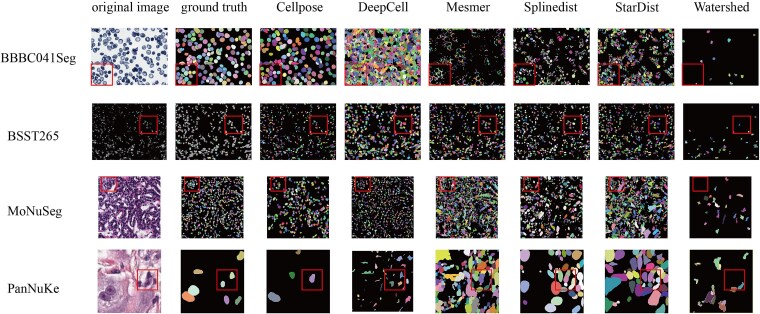
Results of nuclei segmentation methods on image-only datasets. The first column displays the original images, and the second column shows the corresponding segmentation masks. Columns three through the last illustrate the segmentation results obtained using different nuclei segmentation methods. The red rectangle highlights region of interest, enabling detailed evaluation of each algorithm’s performance in nuclei boundary segmentation accuracy and instance segmentation.

Figure 4 and Supplementary Figs 1–4 show visual comparisons of the segmentation results for qualitative assessment. For detailed comparison, we show an image from each dataset, highlighting a region of interest (ROI) with a red rectangle. Close inspection of these key areas reveals that the heterogeneity of the BSST265 dataset visually translates into systematic and diverse error patterns, primarily including: (i) False Positives and Over-segmentation: Texturally complex backgrounds are easily misclassified as cellular signals, causing algorithms to generate numerous noisy fragments; (ii) Under-segmentation and Target Misses: In areas with low contrast or blurry boundaries, algorithms struggle to capture faint signals, leading to missed nuclei; (iii) Instance Merging: In extremely dense clusters of nuclei, the lack of clear separation leads to frequent merging of instances. These qualitative observations align with the metrics observed in the quantitative analysis (e.g. decreased Dice, lower segmentation boundary F1 score), highlighting the severe challenges that real-world domain shifts and complex morphologies pose to algorithmic robustness.

###  Performance comparison of different methods on dataset combining image and sequencing data

In this study, we evaluated nuclear segmentation performance using the mouse brain dataset MSBDS [99], which integrates sequencing information and image. Performance was assessed using instance-level metrics (Precision, Recall, F1-score) and pixel-level accuracy (Dice). Seven mainstream algorithms were tested: Cellpose [81], StarDist [82], DeepCell [90], Splinedist [74], Mesmer [89], Watershed [138] and GeneSegNet [105].

**Figure 5:**
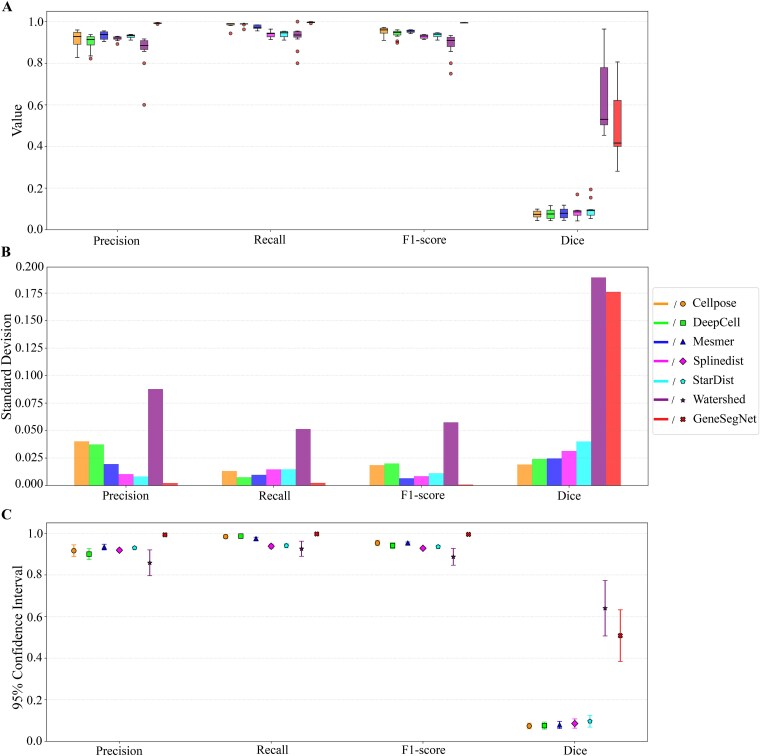
Performance evaluation of seven nuclei segmentation methods on the multi-modal dataset MSBDS integrating image and sequencing data. (A) Shows the segmentation effectiveness. (B) Presents the stability measured by standard deviation. (C) Displays the confidence intervals across all evaluation metrics.

**Figure 6:**
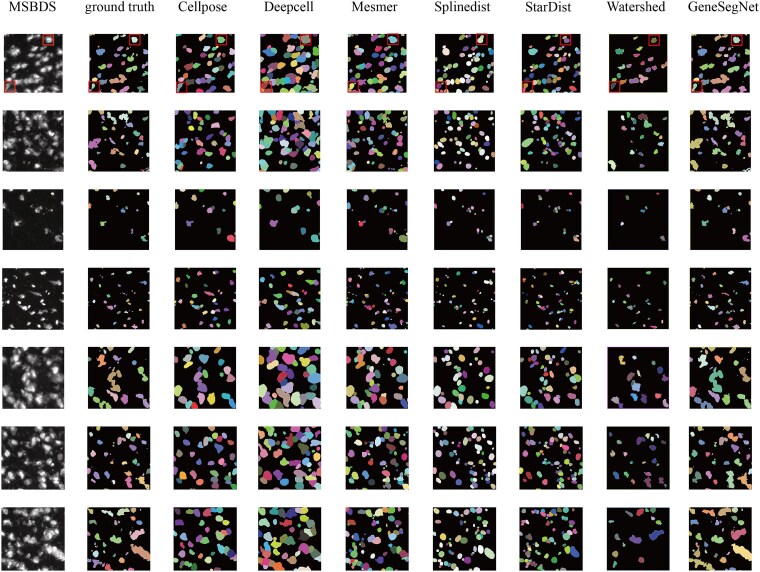
Results of nuclei segmentation methods on the dataset MSBDS integrating image and sequencing data. The first two columns display original images and corresponding ground truth masks, while subsequent columns present results from different segmentation algorithms. A red rectangle highlights a region of interest in the first row to facilitate detailed comparison of boundary accuracy and instance segmentation performance.

As shown in Fig. 5, most methods performed excellently in instance-level recognition, with Precision, Recall, and F1-scores consistently high (0.8–1.0), demonstrating strong nuclei detection and differentiation. However, significant differences emerged in the Dice scores reflecting contour accuracy. Except for Watershed and GeneSegNet, all other algorithms exhibited low Dice values (around 0.1), indicating poor alignment of segmentation masks with ground truth boundaries. GeneSegNet achieved superior performance by integrating imaging and transcriptomic information. To facilitate detailed comparison, we selected identical regions of interest (marked by red rectangles in Fig. 6). The results confirm that GeneSegNet produces more accurate segmentation within these ROIs, whereas other methods exhibited shortcomings like over-segmentation or excessive fragmentation.

###  Quantitative runtime comparison of different methods

To ensure benchmark comparability, all nuclei segmentation experiments were conducted on a unified platform equipped with an Intel Xeon Gold 5117 CPU, 1 TB RAM, a 54 TB hard disk, a Tesla T4 GPU, CentOS Linux 7.9, and CUDA 10.2. We evaluated the computational efficiency of seven methods based on average processing time per image across all datasets (Table 5), alongside instance-level and pixel-level accuracy.

Watershed was the fastest, processing images in 0.01 s on PanNuKe and 0.12 s on MoNuSeg, but its accuracy lagged significantly due to poor adaptation to complex morphologies. In contrast, DeepCell (0.4 s/image on PanNuKe; 5.71 s/image on MoNuSeg) and Mesmer (1.15 s/image on PanNuKe; 14.55 s/image on MoNuSeg) achieved accuracy at higher computational cost. Splinedist offered a favorable speed-accuracy trade-off (PanNuKe: 0.33 s/image; MoNuSeg: 2.89 s/image), with efficiency second only to Watershed and accuracy superior to it, though slightly below DeepCell and Mesmer. Cellpose performed well on PanNuKe (0.4 s/image) with accuracy matching DeepCell, but slowed considerably on MoNuSeg (22.92 s/image). StarDist showed stable processing times (PanNuKe: 1.19 s/image; MoNuSeg: 1.11 s/image) yet suffered accuracy drops due to its reliance on shape priors.

On BBBC041Seg, Cellpose had the highest accuracy but longest time (34.34 s/image), whereas Watershed was fastest (0.04 s/image) but least accurate. StarDist and Splinedist took 1.48 s and 4.15 s, respectively, outperforming Cellpose in speed but not in accuracy. For BSST265, Cellpose and Mesmer required 26.83 s and 14.61 s per image and achieved high recall, though background noise reduced efficiency. StarDist, DeepCell, and Splinedist were faster (ranging from 0.91 to 5.9 s per image) but delivered only moderate accuracy without leading in any metric.

On MSBDS, Watershed and Splinedist remained highly efficient (0.01 s/image; 0.34 s/image) with sufficient accuracy. GeneSegNet delivered superior segmentation by integrating imaging and transcriptomic data, but at a substantial computational cost of 465.2 s per image, illustrating a clear accuracy-efficiency trade-off.

###  Visualization of multi-dimensional performance of cell segmentation methods

**Figure 7:**
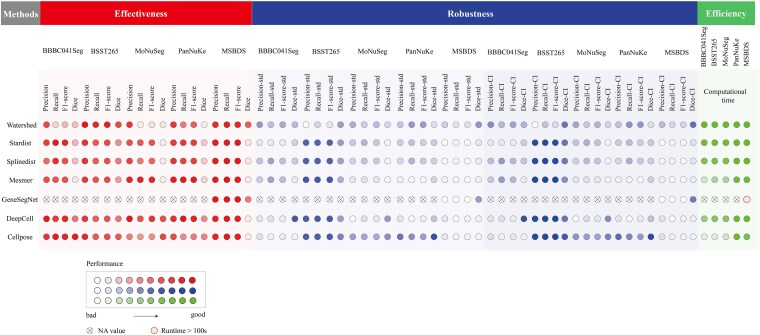
Performance visualization results of seven representative nuclei segmentation methods assessed on effectiveness (red), robustness (blue), and efficiency (green). For all colored dots, a darker shade denotes better performance. A cross indicates the method is not applicable (NA), and a red-bordered circle signifies a runtime over 100 s.

To provide an integrated visualization of all algorithms’ performance, we developed a performance heatmap (Fig. 7) based on a 3D ‘Effectiveness–Robustness–Efficiency’ framework, using RGB color channels for intuitive multi-dimensional representation. The red channel indicates effectiveness, derived from the average of Precision, Recall, F1-score, and Dice—darker red indicates better segmentation. The blue channel represents robustness, calculated from the stability (standard deviation and confidence intervals) of metrics across datasets—darker blue implies higher consistency and adaptability. The green channel corresponds to efficiency, based on average runtime across datasets—darker green denotes lower computational cost. In all channels, darker shades represent good performance, while lighter shades indicate poor performance, allowing clear visual comparison.

Different nuclear segmentation methods demonstrate varying performance levels across datasets. On BBBC041Seg with regular-shaped nuclei, Cellpose excelled in all metrics using its flow-based morphological model. For datasets with irregular or adherent nuclei (e.g. MoNuSeg, PanNuKe), DeepCell and Mesmer led in instance-level metrics (Precision, Recall, F1-score). The highly diverse BSST265 dataset challenged most algorithms; only Cellpose and Watershed maintained reasonable pixel-level accuracy. On MSBDS, most methods performed well in instance recognition, and GeneSegNet delivered superior segmentation by integrating imaging and transcriptomics, though with higher computational cost.

In practical applications, it is essential to select an appropriate nuclear segmentation method based on the specific requirements and characteristics of the dataset to achieve optimal segmentation results and efficiency. For tissue image datasets with complex features such as atypical nuclei or nuclear adhesion (e.g. PanNuKe), DeepCell or Mesmer are recommended due to their strong generalization ability without relying on fixed shape priors, ensuring high instance-level segmentation performance. For datasets with regular nuclear morphologies (e.g. BBBC041Seg), Cellpose is preferable for its superior pixel-level accuracy achieved through edge fitting. It is recommended to refer to validation results from similar datasets and aim for a tailored ‘task–data–algorithm’ match rather than blindly pursuing a universally optimal solution.

###  Visualization of multi-dimensional attributes and scenario-adaptive recommendation for cell segmentation methods

To further clarify the methodological positioning of evaluated algorithms and facilitate practical application, two complementary visualizations were developed.

**Figure 8:**
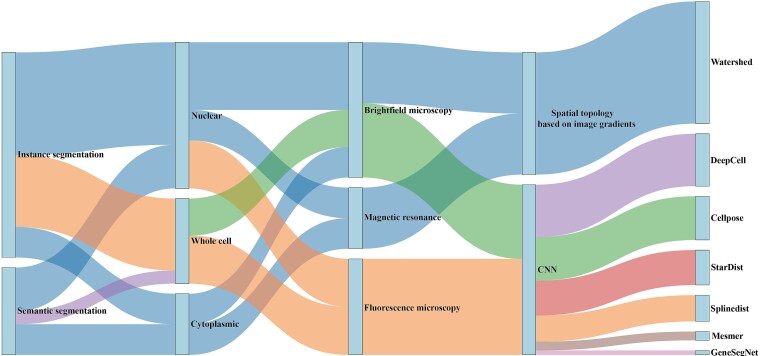
A Sankey diagram mapping the seven evaluated tools onto the proposed ‘task-target-modality-method’ taxonomy. The flow illustrates the methodological basis (from classical to deep learning) and the operational scope (modality and target) of each algorithm.

Figure 8 presents a Sankey diagram mapping the seven evaluated tools (Cellpose, StarDist, DeepCell, Splinedist, Mesmer, Watershed, GeneSegNet) onto the proposed ‘task-target-modality-method’ four-dimensional classification system. The diagram uses flow paths with varying widths to illustrate the dual attributes of each algorithm: (i) the methodological basis (from classical techniques to deep learning-based approaches); (ii) the operational scope, including compatible imaging modalities (bright-field, fluorescence, spatial omics-integrated) and segmentation targets. This visualization intuitively illustrates the structural relationships between algorithms and the classification framework, enabling researchers to quickly grasp the inherent characteristics and applicable boundaries of each method.

**Figure 9:**
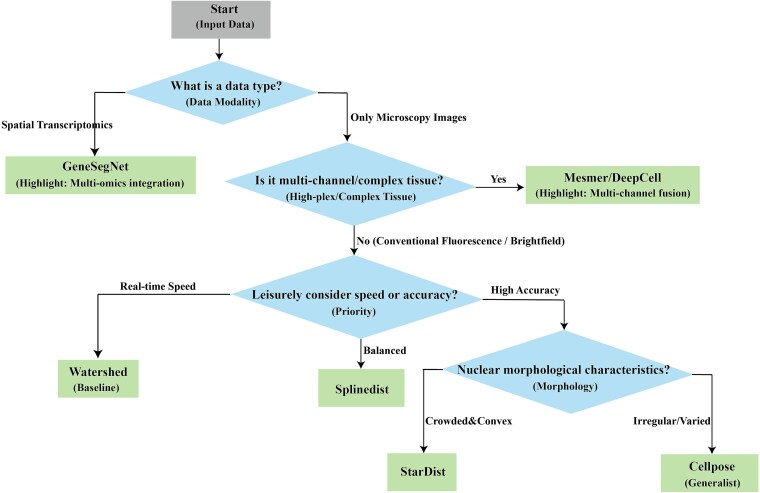
A decision-tree recommendation guide for users and developers. The flowchart assists researchers in selecting the optimal segmentation tool based on data modality (e.g. spatial omics versus standard imaging), tissue complexity, and specific task requirements (e.g. speed versus accuracy trade-offs).

Figure 9 complements the quantitative performance results with a decision-tree recommendation guide for users and developers. The flowchart is constructed based on three key practical criteria derived from experimental findings: (i) data modality (e.g. standard imaging versus spatial omics-integrated data like MSBDS); (ii) tissue/ sample complexity (e.g. regular nuclear morphology versus heterogeneous tissue versus adherent nuclei); (iii) core task requirements (e.g. prioritizing speed versus pixel-level accuracy versus balanced instance-level performance). By following the decision nodes, researchers can efficiently identify the optimal segmentation tool tailored to their specific experimental scenarios, bridging the gap between benchmark results and real-world application.

##  Discussion

This study presents a systematic investigation into cell segmentation technology, encompassing a review of methodologies and the development of a distinct classification system, with a focus on nuclear segmentation for experimental validation and analysis. The review aims to offer practical guidance for method selection, particularly for new researchers, and to address gaps in existing literature.

First, we conducted a comprehensive survey and introduced a systematic classification of computational methods for cell segmentation, establishing a coherent conceptual framework that spans traditional techniques, traditional machine learning, and deep learning approaches. Specifically, for deep learning models, we proposed a taxonomy based on task-driven and data-driven criteria, and further constructed a four-dimensional classification system organized by ‘task-target-modality-method,’ covering various segmentation targets (nuclei, cytoplasm, organelles, whole cells) and imaging modalities (bright-field, fluorescence).

Second, we established a technical benchmark for nuclear segmentation methods through carefully designed datasets and rigorous evaluation. The datasets include two scenarios: nuclei-only images and nuclei integrated with sequencing data. We evaluated seven algorithms based on three core dimensions: effectiveness, robustness, and efficiency, enabling a quantitative comparison of each method’s performance and applicability.

Our evaluation suggests several key insights: algorithm performance is context-specific. Cellpose, DeepCell, and Mesmer each excel in particular scenarios—regular morphology images or complex tissue samples. Two major challenges were identified: insufficient pixel-level segmentation accuracy in complex environments, and performance instability when handling highly diverse data (e.g. BSST265). Furthermore, deep learning methods (e.g. DeepCell, GeneSegNet) consistently outperform traditional algorithms like Watershed in accuracy and robustness. Integrating multimodal data, such as sequencing information (e.g. GeneSegNet), further improves boundary identification.

Based on these findings, we suggest three future directions: (i) improving boundary detection for higher pixel-level accuracy in challenging scenarios; (ii) enhancing feature extraction modules to generalize across diverse data; and (iii) developing intelligent matching strategies between data and algorithms. Innovations may involve attention mechanisms, adaptive architectures, and multi-model collaboration, advancing nuclear segmentation for applications in pathological diagnosis and drug development.

In summary, this study clarifies the principles, applicability, and performance of various methods through in-depth analysis and benchmarking. These contributions enhance the understanding of nuclear segmentation and support more reliable bioinformatics analysis. Future work should focus on creating automated, precise, efficient, and adaptive segmentation methods. With continued progress in deep learning, more mature solutions are likely to play an expanding role in biomedical research and clinical practice.

##  Methods

###  Datasets

To ensure a fair evaluation, we utilized five datasets: four image-only types—PanNuKe [147], MoNuSeg [148], BBBC041Seg [149], and BSST265 [150], as well as MSBDS [99], which integrates image with sequencing information.

The PanNuKe dataset contains 901 images (256 × 256px, 40× magnification) from whole slide images, covering 19 tissue types with 189 744 annotated nuclei across five categories. MoNuSeg includes 30 images (1000 × 1000px) from TCGA whole slides across seven organs, exhibiting staining variations due to multi-center sourcing, with over 21 000 expert-annotated nuclei. BBBC041Seg comprises 1328 Giemsa-stained blood smear images (1600 × 1200px) with 93 539 leukocyte nuclei, offering high contrast suitable for deep learning. BSST265 contains 79 antibody fluorescence and DAPI-stained images with 7813 nuclei from diverse clinical and cell line sources, designed to support tumor pathology studies. MSBDS integrates image and sequencing data from mouse brain, acquired via Stereo-seq [156], with subcellular resolution. This technology produces spatial transcriptomic data, where each spatial unit is represented by a high-dimensional vector of gene expression counts. For computational efficiency, the data were processed into non-overlapping 225 × 225px patches. Key attributes of all datasets are summarized in Table 3. All annotations originate from their respective public benchmarks.

**Table 3:** Overview and characteristics of the experimental datasets.

Dataset	Type of imaging (microscopic technique)	Dyeing patterns	Size	Number of images	Annotated method	Introduction
PanNuKe	Bright field microscope	H&E	256 × 256	7901	Automated annotation is used and quality control is performed by clinical pathologists	19 tissue types, five cell types
MoNuSeg	Bright field microscope	H&E	1000 × 1000	30	The cell nuclei boundaries are annotated by a pathologist after annotation is completed through Aperio ImageScope	Seven tissue types
BBBC041Seg	Bright field microscope	Giemsa	1600 × 1200	1328	Use semi-automatic tools and manually validated cell nuclei	Blood cells (mainly white blood cells)
BSST265	Fluorescence microscope	DAPI	Multiple sizes (including 1225 × 914, 1280 × 1024, etc.)	79	It was annotated and reviewed by pathologists and disease specialists	Cell nuclei from various cell types and tissues
MSBDS	Spatial transcriptome (sequencing + optical microscopy)	DAPI	20 000 × 20 000	1	ImageJ and LabelMe software were used to complete the nuclear annotation and manual verification to ensure the quality of annotation	Brain sections from adult mice

Notably, microscopy and spatial transcriptomics data differ in principle, sample requirements, and application. Microscopy captures morphological information directly through optical imaging, yet is limited by diffraction-induced blurring. Spatial transcriptomics computationally reconstructs gene expression spatiality using *in situ* RNA capture with barcoded probes. It requires fresh-frozen or specially processed FFPE samples, a requirement that limits its clinical applicability. While microscopy allows real-time morphological observation, spatial transcriptomics reveals molecular heterogeneity. However, the latter requires integration with single-cell data for annotation and lacks dynamic monitoring capability. The two modalities are complementary: microscopy offers structural context for transcriptomic data, which in return provides functional interpretation. A comparative summary of both data types and their impact on segmentation method suitability is provided in Table 4.

**Table 4:** Differences between microscopic image data and spatial transcriptomics data.

Comparison dimension	Brightfield microscopy	Fluorescence microscopy	Spatial transcriptomics
Imaging mechanism	Imaging based on transmitted light absorption differences	Imaging by fluorescence emission of fluorophores upon excitation	mRNA capture and sequencing via molecular probes
Typical platforms	Ordinary optical microscopes	Confocal/Light-sheet fluorescence microscopy	Stereo-seq, 10X Visium, Slide-seq
Sample requirements	Paraffin embedding/HE/Giemsa staining	Fixed or live cells/fluorescence labeling (e.g. DAPI)	Fresh-frozen tissue/permeabilization treatment
Resolution	~200 nm (optical diffraction limit)	~200 nm (optical diffraction limit)	Determined by probe density (e.g. Stereo-seq ~500 nm)
Data type	Multi-channel RGB images	Single/multi-channel high-contrast grayscale images	Gene expression matrix and spatial coordinates
Core information	Morphological structures (cell/tissue arrangement)	Molecular localization (protein/nucleic acid distribution)	Spatial mapping of gene expression
Signal specificity	Regional staining (e.g. nuclear/cytoplasmic)	Molecular-level specificity (targeted labeling)	Whole-transcriptome coverage
Advantages	Low cost and rapid identification of pathological structure	High specificity, live-cell dynamic tracking	Decoding spatial heterogeneity
Key challenges	Uneven staining, blurred boundaries	Fluorescence crosstalk, photobleaching	Low resolution, sparse data
Segmentation tasks	Nuclear /cytoplasm/whole-cell segmentation	Nuclear/cytoplasm/whole-cell segmentation	Nuclear /whole-cell segmentation
Typical applications	Pathological diagnosis, blood smear analysis	Protein sub-cellular localization, organelle dynamics	Developmental biology, tumor microenvironment studies

**Table 5:** The running time of a single image.

Methods datasets	Cellpose	StarDist	DeepCell	Splinedist	Mesmer	Watershed	GeneSegNet
BBBC041Seg	34.34 s	1.48 s	9.90s	4.15 s	23.55 s	0.04 s	NA
BSST265	26.83 s	0.91 s	5.90s	2.90s	14.61 s	0.12 s	NA
MoNuSeg	22.92 s	1.11 s	5.71 s	2.89 s	14.55 s	0.12 s	NA
PanNuKe	0.40s	0.19 s	0.40s	0.38 s	1.15 s	0.01 s	NA
MSBDS	3.47 s	0.20s	0.54 s	0.34 s	1.30s	0.01 s	465.20s

###  Model architecture and default parameters

For benchmark comparisons, all methods were evaluated under standardized configurations. Watershed was implemented using the default OpenCV parameters. Deep learning methods employed their respective pre-trained models with hyper-parameters strictly following the original publications.

Cellpose used the pre-trained model with the configuration: models.Cellpose (gpu = True, model_type = ‘nuclei’).

StarDist employed pre-trained models adapted for different imaging modalities: StarDist2D.from_pretrained (‘2D_versatile_fluo’) for fluorescence and StarDist2D.from_pretrained (‘2D_versatile_he’) for brightfield images.

Splinedist was configured with the 6-control-point model to balance contour detail and smoothness. The fluorescence and brightfield configurations were SplineDist2D (None, name = ‘splinedist_6,’ basedir = ‘fluo/models’) and SplineDist2D (None, name = ‘splinedist_6,’ basedir = ‘he/models’), respectively.

DeepCell utilized a pre-trained model, with its specific parameter configuration set as ‘app = NuclearSegmentation ()’.

Mesmer adopted the pre-trained model, configured with app = Mesmer () for initialization, followed by prediction via app.predict (X_train, image_mpp = 0.5, compartment = ‘nuclear’).

GeneSegNet employed a pre-trained model, with its specific parameter configuration set as:

“model = models.GeneSegModel (

gpu = gpu,

pretrained_model = pretrained_model

).”

###  Evaluate metrics

In this study, all algorithms perform instance segmentation, and their output object masks are directly used for performance evaluation.

Effectiveness focuses on evaluating whether a model can accurately and reliably segment target regions in images to achieve the expected segmentation goals. For this, we use Precision, Recall, F1-score, and Dice coefficient as quantitative metrics [151, 157].

Intersection over Union (IoU) quantifies the overlap between a prediction and its closest ground truth object:


(1) \begin{equation*} \mathrm{IoU}=\frac{P\cap G}{P\cup G} \end{equation*}

Where P denotes the prediction and G the ground truth, a ground truth object is considered correctly segmented if the IoU between it and its closest prediction exceeds 0.5.

Precision and Recall are defined as:


(2) \begin{equation*} \mathrm{Precision}=\frac{TP}{TP+ FP} \end{equation*}

(3) \begin{equation*} \mathrm{Recall}=\frac{TP}{TP+ FN} \end{equation*}

where TP is the count of True Positives, FP is the count of False Positives, and FN is the count of False Negatives.

The F1-score, the harmonic mean of Precision and Recall, evaluates their balance.


(4) \begin{equation*} \mathrm{F}1-\mathrm{score}=2\times \frac{Precision\times Recall}{Precision+ Recall} \end{equation*}

In addition, a pixel-level metric is also introduced to assess segmentation quality by comparing predicted and ground truth masks.


(5) \begin{equation*} \mathrm{Dice}=\frac{2\times TP}{2\times TP+ FP+ FN} \end{equation*}

This scheme evaluates both the distinction of individual instances (via instance-level metrics) and the precision of segmentation boundaries (via pixel-level metrics). This design aligns with the evaluation requirements of instance segmentation tasks.

Robustness evaluates the ability of nuclear segmentation methods to maintain stable performance across varying inputs, such as noise, imaging conditions, and sample diversity. In this study, it specifically refers to consistency in cross-dataset performance. We quantify robustness using the standard deviation and confidence intervals of effectiveness metrics. A small standard deviation indicates stable results across conditions, while a narrow confidence interval reflects high estimation reliability. Both conditions must be met simultaneously to demonstrate strong robustness. By integrating these measures, we compare the robustness of different segmentation methods.

Additionally, we use the actual runtime of the algorithms as the metric for efficiency to comprehensively evaluate the performance of each method.

**Key Points**

- We reviewed 68 cell segmentation tools, categorized as traditional, machine learning-based, or deep learning-based, and analyzed their strengths and limitations across algorithms, key aspects, and tools. In the deep learning section, we introduce a classification framework that categorizes methods as task-oriented or data-oriented. Furthermore, we establish a systematic ‘task-target-modality-method’ taxonomy to comprehensively organize cell segmentation approaches.
- We curated five benchmark datasets to support nuclei segmentation research. These include four conventional microscopy image collections and one novel set that integrates sequencing with image data. We evaluated seven representative algorithms using these datasets.
- We found deep learning methods outperformed traditional algorithms in accuracy and robustness. Additionally, integrating multimodal data, such as image and sequencing data, can improve nuclear boundary recognition.

## Supplementary Material

Supplementary_material_bbag066

## Data Availability

All the five independent datasets used for benchmarking various nuclei segmentation methods in this study are available at http://jianglab.org.cn/cell_segmentation_download/.
