# Supplementary material for: Systematic evaluation of computational methods for cell segmentation
Source: Brief Bioinform. 2026 Feb 24;27(1):bbag066. doi: 10.1093/bib/bbag066 (PMC12931453; doi:10.1093/bib/bbag066)
Supplement: Supplementary_material_bbag066 [file supplementary_material_bbag066.docx]

**Supplementary figure legends**


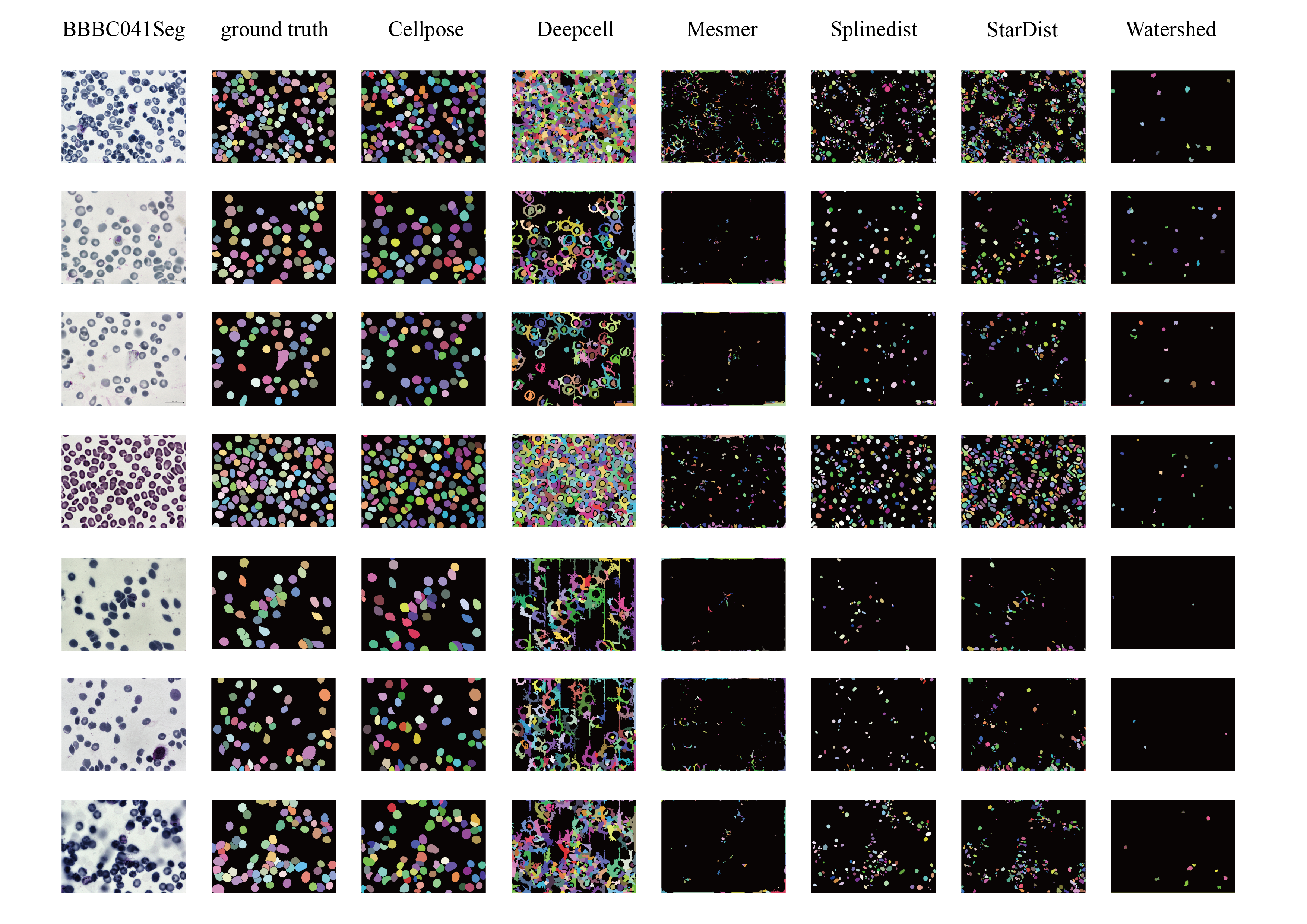


Supplementary Figure 1. Results of nuclei segmentation methods on image-only dataset BBBC041Seg. The first column displays the original images, and the second column shows the corresponding segmentation masks. Columns three through the last show the segmentation results obtained using different nuclei segmentation methods. (ALT Text: Multi-column comparison for BBBC041Seg, showing original nuclei images, reference masks and varied algorithm segmentation results.)


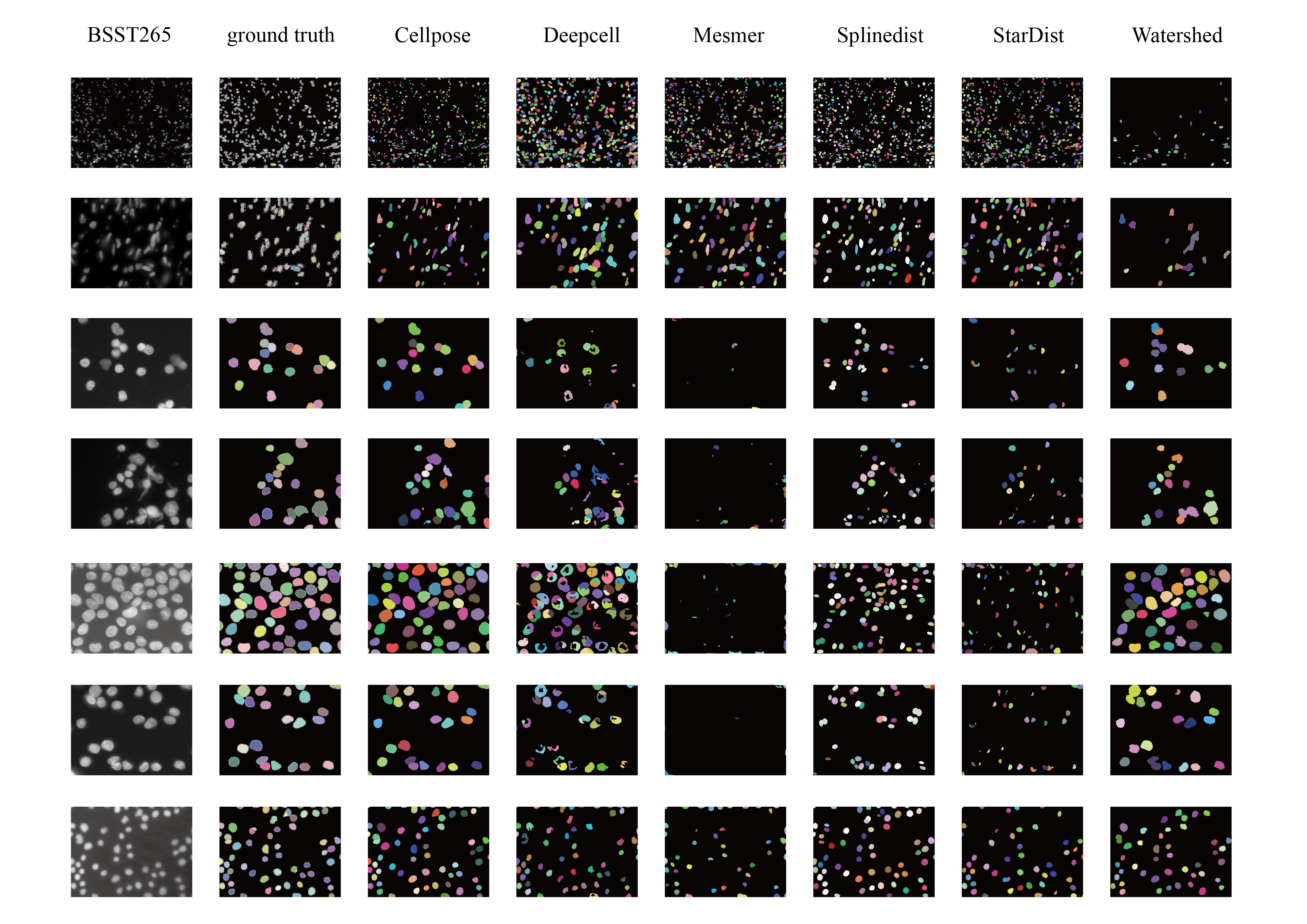


Supplementary Figure 2. Results of nuclei segmentation methods on image-only dataset BSST265. The first column displays the original images, and the second column shows the corresponding segmentation masks. Columns three through the last show the segmentation results obtained using different nuclei segmentation methods. (ALT Text: Multi-column comparison for BSST265, showing original nuclei images, reference masks and varied algorithm segmentation results.)


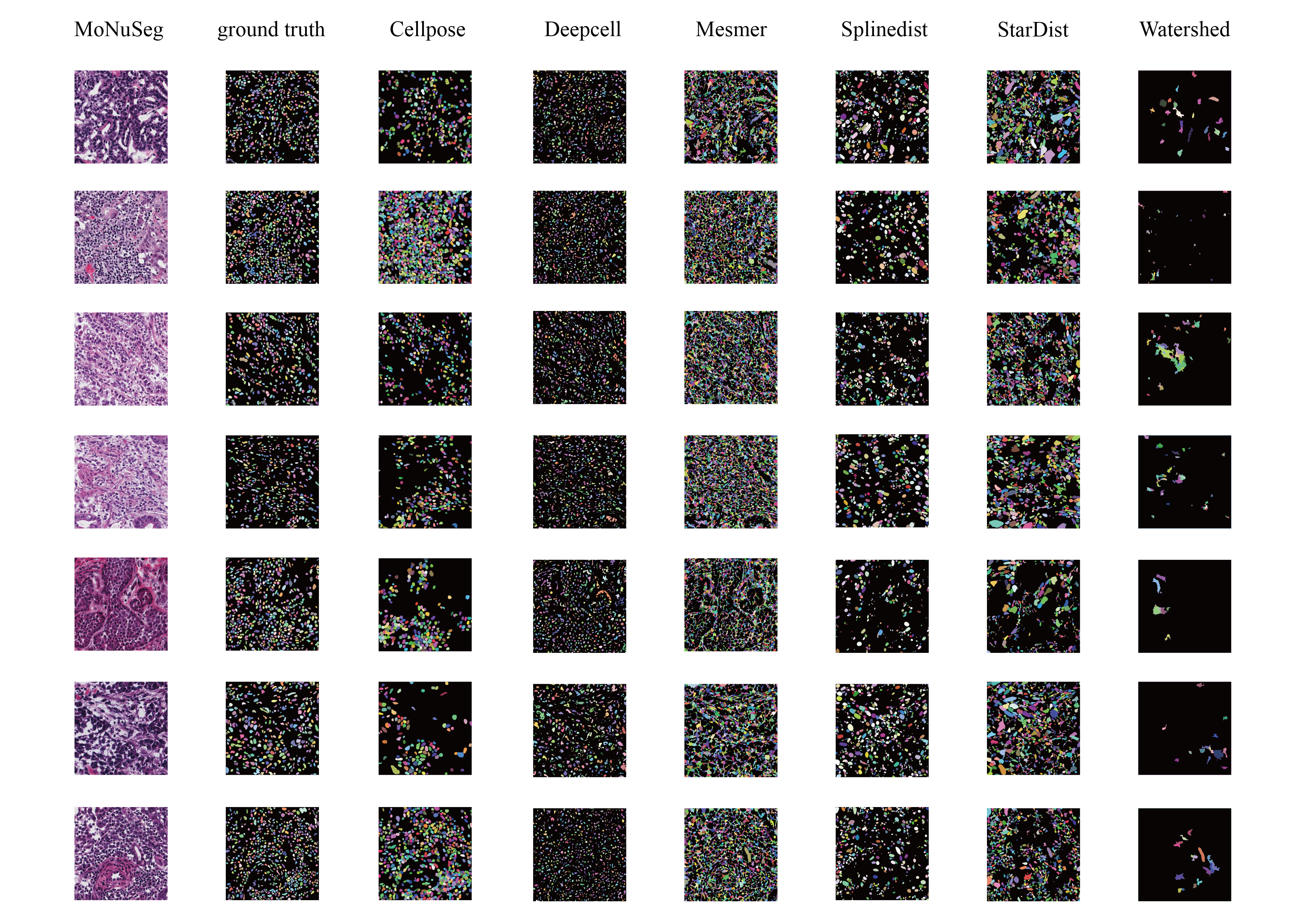


Supplementary Figure 3. Results of nuclei segmentation methods on image-only dataset MoNuSeg. The first column displays the original images, and the second column shows the corresponding segmentation masks. Columns three through the last show the segmentation results obtained using different nuclei segmentation methods. (ALT Text: Multi-column comparison for MoNuSeg, showing original nuclei images, reference masks and varied algorithm segmentation results.)


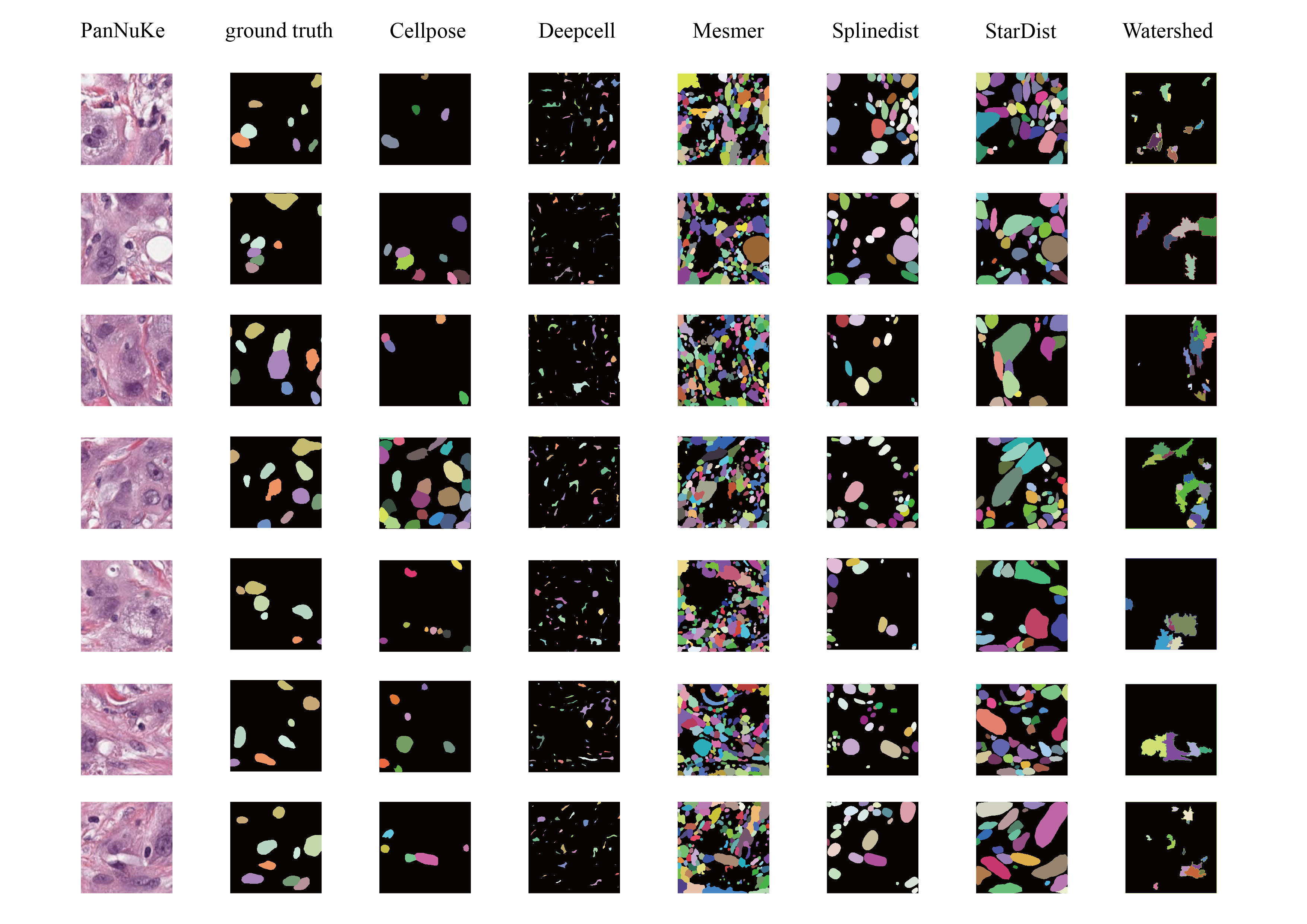


Supplementary Figure 4. Results of nuclei segmentation methods on image-only dataset PanNuKe. The first column displays the original images, and the second column shows the corresponding segmentation masks. Columns three through the last show the segmentation results obtained using different nuclei segmentation methods. (ALT Text: Multi-column comparison for PanNuKe, showing original nuclei images, reference masks and varied algorithm segmentation results.)
